# Human Group IIA Phospholipase A_2_—Three Decades on from Its Discovery

**DOI:** 10.3390/molecules26237267

**Published:** 2021-11-30

**Authors:** Kieran F. Scott, Timothy J. Mann, Shadma Fatima, Mila Sajinovic, Anshuli Razdan, Ryung Rae Kim, Adam Cooper, Aflah Roohullah, Katherine J. Bryant, Kasuni K. Gamage, David G. Harman, Fatemeh Vafaee, Garry G. Graham, W. Bret Church, Pamela J. Russell, Qihan Dong, Paul de Souza

**Affiliations:** 1 School of Medicine, Western Sydney University, Campbelltown, NSW 2560, Australia; 17432636@student.westernsydney.edu.au (T.J.M.); shadma.fatima@inghaminstitute.org.au (S.F.); adam.cooper@health.nsw.gov.au (A.C.); aflah.roohullah@health.nsw.gov.au (A.R.); p.desouza@westernsydney.edu.au (P.d.S.); 2 Ingham Institute of Applied Medical Research, Liverpool, NSW 2170, Australia; mila.sajinovic@inghaminstitute.org.au (M.S.); 18525218@student.westernsydney.edu.au (A.R.); 3 School of Biotechnology and Biological Sciences, University of New South Wales (UNSW Sydney), Sydney, NSW 2052, Australia; f.vafaee@unsw.edu.au; 4 School of Pharmacy, Faculty of Medicine and Health, University of Sydney, Sydney, NSW 2006, Australia; rkim2691@alumni.sydney.edu.au (R.R.K.); bret.church@sydney.edu.au (W.B.C.); 5 Liverpool Cancer Therapy Centre, Liverpool Hospital, Liverpool, NSW 2170, Australia; 6 School of Photovoltaic and Renewable Energy Engineering, UNSW Sydney, Sydney, NSW 2052, Australia; katherine.bryant@unsw.edu.au; 7 School of Science, Western Sydney University, Campbelltown, NSW 2560, Australia; 19243506@student.westernsydney.edu.au (K.K.G.); d.harman@westernsydney.edu.au (D.G.H.); 8 UNSW Data Science Hub, UNSW Sydney, Sydney, NSW 2052, Australia; 9 Department of Clinical Pharmacology, St Vincent’s Hospital Sydney, Darlinghurst, NSW 2010, Australia; g.graham@unsw.edu.au; 10 School of Medical Sciences, UNSW Sydney, Sydney, NSW 2052, Australia; 11 Australian Prostate Cancer Research Centre—QUT, Brisbane, QLD 4102, Australia; Pamela.russell@qut.edu.au; 12 Chinese Medicine Anti-Cancer Evaluation Program, Greg Brown Laboratory, Central Clinical School and Charles Perkins Centre, The University of Sydney, Sydney, NSW 2006, Australia; qihan.dong@sydney.edu.au; 13 School of Medicine, UNSW Sydney, Sydney, NSW 2052, Australia

**Keywords:** eicosanoid, drug development, chronic inflammation, cancer, prostaglandin

## Abstract

Phospholipase A_2_ (PLA_2_) enzymes were first recognized as an enzyme activity class in 1961. The secreted (sPLA_2_) enzymes were the first of the five major classes of human PLA_2_s to be identified and now number nine catalytically-active structurally homologous proteins. The best-studied of these, group IIA sPLA_2_, has a clear role in the physiological response to infection and minor injury and acts as an amplifier of pathological inflammation. The enzyme has been a target for anti-inflammatory drug development in multiple disorders where chronic inflammation is a driver of pathology since its cloning in 1989. Despite intensive effort, no clinically approved medicines targeting the enzyme activity have yet been developed. This review catalogues the major discoveries in the human group IIA sPLA_2_ field, focusing on features of enzyme function that may explain this lack of success and discusses future research that may assist in realizing the potential benefit of targeting this enzyme. Functionally-selective inhibitors together with isoform-selective inhibitors are necessary to limit the apparent toxicity of previous drugs. There is also a need to define the relevance of the catalytic function of hGIIA to human inflammatory pathology relative to its recently-discovered catalysis-independent function.

## 1. Introduction

**Figure 1:**
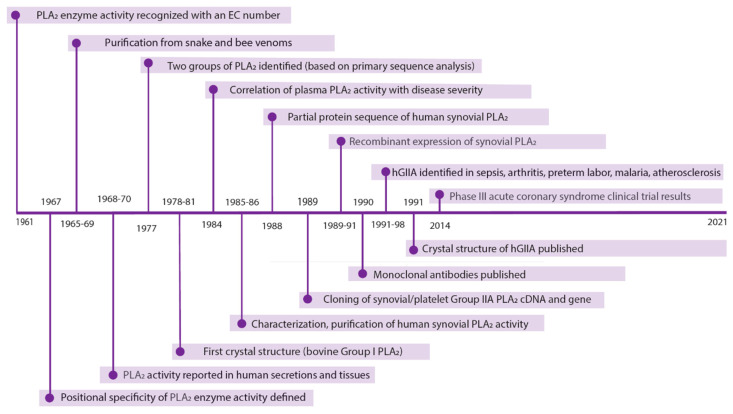
Timeline of major discoveries in hGIIA drug development.

Phospholipase A_2_ (PLA_2_) enzyme reactions were formally classified with an Enzyme Commission number (EC 3.1.1.4) in 1961 [1] and have been the subject of intense study since then. A literature search on “phospholipase A2” in 2021 retrieved over 28,700 publications [2] in the 60 years since the enzyme activity was defined. Growth in publication numbers was exponential up until the early 1990s and has grown linearly in the subsequent 30 years to reach a current rate of ~1700 publications per year in 2020–2021. The discovery that PLA_2_ enzyme activity was a property shared by a large superfamily of proteins led to the codification of the enzyme class in a nomenclature system by Prof. Ed Dennis and colleagues, based on structural and functional features of the enzymes [3,4]. This system recognized five classes of active enzyme *viz*. secreted PLA_2_s (sPLA_2_s) cytosolic PLA_2_s, (cPLA_2_s), calcium-independent PLA_2_s, the platelet activating factor acetylhydrolases and the lysosomal PLA_2_s. To date, there are nine sPLA_2_ enzymes expressed in humans, classified as groups IB, IIA, IID, IIE, IIF, III, V, X and XIIA. A brief and by no means exhaustive timeline of some major discoveries related to human group IIA phospholipase A_2_ (human PLA2GIIA, human sPLA_2_-IIA, hGIIA), the subject of this review, is shown in Figure 1.

PLA_2_ enzymes are defined by their positional specificity for cleavage of the *sn*-2 ester bond of phospholipids to release free fatty acid and lysophospholipid as reported in extracts of the bovine adrenal medulla [5]. The enzymes were first purified and characterized from snake and bee venoms [6,7,8,9] with the first crystal structure of a PLA_2_, isolated from bovine pancreas, published in 1978 [10,11]. The comparison of the primary sequence of PLA_2_ from several species led to the separation of these enzymes into two groups distinguished by structural features, with group I comprised of enzymes from elapid snakes and the pancreas and group II derived from crotalid and viper snakes [12].

In humans, PLA_2_ activity, was first reported in pancreatic juices [13] and cerebrospinal fluid and brain tissue [14,15]. Over a decade later, during which time enzyme activity assays were refined [16,17] PLA_2_ activity was detected in plasma from patients with hypotension and septicemia, providing the first evidence that plasma PLA_2_ activity correlated with severity of disease [18]. The characterization [19], purification [20] and partial protein sequence [21] of a PLA_2_ from the synovial fluid of patients with rheumatoid arthritis and from platelets [22] followed over the next four years. This information facilitated the cloning of the gene and recombinant expression of this enzyme [22,23,24]. Comparison of the predicted PLA_2_ sequence with the two groups of snake enzyme sequences confirmed the synovial enzyme as a group II PLA_2_ and with the discovery of several homologous human gene sequences, this enzyme became referred to as group IIA PLA_2_ [25]. Selective monoclonal antibodies and their use to identify and quantify hGIIA in plasma and in tissues and to inhibit its activity, very quickly followed [26,27,28,29,30,31,32].

The structure of hGIIA, published in 1991 [33], led to the development and clinical trial of highly potent active-site inhibitors of hGIIA catalytic activity. Despite these efforts over the following 30 years, there are no hGIIA inhibitors approved for clinical use. Phase III clinical trials in patients with acute coronary syndrome were halted due to safety concerns [34]. This review will explore the hGIIA literature in an effort to shed light on features of hGIIA function that may account for this lack of success and discuss potential directions for further research that may be of benefit in realizing the potential clinical utility of targeting hGIIA function.

## 2. Relevant Discoveries, 1991–2014

Progress in the phospholipase A_2_ field between 1991 and 2014 has been extensively reviewed by many groups in both general and specific areas with some examples referenced here [35,36,37,38,39]. Key developments of relevance to hGIIA function in inflammation were the discovery that the group IIA PLA_2_ gene is non-functional in certain mouse strains, demonstrating that the enzyme has no universally-essential physiological function in mammals and raising the possibility that enzyme function may be influenced by background genotype. The finding also raised practical difficulties in generating isogenic in vivo models where sPLA_2_ expression could be ablated. Transgenic overexpression of hGIIA in mice provided some insight into the effect of upregulation of the enzyme on phenotype. However it was clear from the data emerging from cell culture studies and from serum hGIIA measurements that the effect of aberrant expression of hGIIA was influenced by cell type, quantitative level of expression and species differences. Robust translation of preclinical findings to clinically-relevant settings was therefore difficult. Nonetheless, developments in in vivo gene deletion technologies, together with the development of a model of inflammatory arthritis that enabled a pathology resembling human arthritis to be induced in any mouse strain by adoptive transfer of serum demonstrated that group IIA sPLA_2_ was proinflammatory in this model, while the related group V sPLA_2_ was suppressive of inflammation [40]. Further, the closely related homolog of group IIA sPLA_2_, group IID, has anti-inflammatory effects in lymphoid tissue, resolving hapten-dependent contact dermatitis in mice [41]. 

The finding that structurally-related sPLA_2_ enzymes could have opposing effects in a model of inflammation has significant implications for the development of clinically effective inhibitors of hGIIA enzyme activity. The active site and catalytic mechanism of sPLA_2_ enzymes are conserved between isoforms within species and between species. The inhibitor of hGIIA catalysis, trialed in the clinic, Varespladib [35], despite being highly selective for sPLA_2_ over other PLA_2_ enzymes, is non-selective for hGIIA over other human secreted enzymes, also potently inhibiting group V sPLA_2_ (hGV), group X sPLA_2_ (hGX), group IB, and group IIE enzymes [42], note: Varespladib is referred to as compound B in this study]. Thus, given there are nine sPLA_2_ enzymes in humans, selectivity within this group will be an important requirement of effective inhibition of hGIIA-mediated inflammation.

## 3. hGIIA Function

As described above, the functional impact of hGIIA expression is highly dependent on cell and tissue type and varies substantially depending on the biochemical context of its expression. In a physiological context, hGIIA has a well described role in host defense mediated by its catalytic activity and substrate preference for bacterial membrane phospholipids phosphatidylglycerol and phosphatidylethanolamine [43,44]. However hGIIA function in a pathological context is less clear due to its almost ubiquitous upregulation at organ sites of both chronic and acute inflammation and its implied but poorly-defined role in directly generating pro-inflammatory lipid mediators. hGIIA, in cell culture and in vitro experiments, produces lipid mediators such as the potent signaling and only water-soluble lipid mediator, lysophosphatidic acid (LPA) [45] and arachidonic acid (AA) [39]. AA is metabolized further by cyclooxygenase, lipoxygenase and cytochrome P450 enzymes to produce a range of eicosanoid autocrine and paracrine signaling molecules [39]. LPA and eicosanoids have various physiological roles acting through G-protein coupled receptors (GPCRs) [39,45]. 

With the exception of physiological host responses to localized inflammation resulting from injury or minor infection and in childbirth, inflammatory mediator production is not the dominant physiological role of hGIIA. Its ability to function as a phospholipase on the membranes of normal cells is somewhat limited. The highly cationic nature (pI > 10.5) of hGIIA [46], contributes to its low affinity for the zwitterionic lipid phosphatidylcholine (PC), which comprises the majority of the outer leaflet of the phospholipid membrane [47]. Several of its preferred substrates; phosphatidylserine (PS), phosphatidylethanolamine (PE) and phosphatidylglycerol (PG) [48], are present at higher concentrations on the inner leaflet of the membrane than the outer leaflet. In addition, intracellular cytosolic calcium levels are far lower than the millimolar concentrations required for hGIIA catalytic activity. Therefore under physiological conditions, hGIIA generally does not contribute to AA production through enzymatic activity on the plasma membrane of viable cells [49].

Plasma membrane asymmetry, that protects the cell from hGIIA catalytic activity, is lost during apoptosis, allowing hGIIA-mediated hydrolysis of the lipid bilayer [50]. This indicates a potential additional physiological role for hGIIA, involving the removal of cellular debris, and may also exacerbate certain pathologies where lipid asymmetry is lost.

### 3.1. hGIIA Function in Host Defense

The physiological functions of hGIIA occur in the extracellular milieu and are facilitated by electrostatic interactions generated by its positive charge (+14), as most of its preferred substrates are anionic. As mentioned above, the best characterized role of hGIIA is in host defense [43,44]. First identified in the 1970s [51,52,53], hGIIA acts as a first line of defense against gram-positive bacteria [54,55], attracted by the negatively charged teichoic and lipoteichoic acids in the peptidoglycan cell wall [56,57,58,59]. hGIIA has the highest bactericidal activity of any sPLA_2_ [60] and is most potent during the logarithmic phase of bacterial growth [57]. During this phase, hGIIA can penetrate the dividing cell wall and hydrolyze its preferred substrates PG and PE in the plasma membrane, causing lysis. Gram-negative bacteria have a thinner peptidoglycan layer with an outer membrane coated with positively charged lipopolysaccharide (LPS), inhibiting hGIIA hydrolysis of the anionic plasma membrane [61]. Factors such as bactericidal/permeability increasing proteins (BPI) and lysozyme can disrupt the cell wall, allowing for hGIIA mediated lysis [62,63]. The presence of hGIIA in secretory glands and cells such as tear ducts, salivary glands, the prostate and seminal vesicles, Paneth cells, the lactating breast and gestational tissues also indicate that host defense is the primary physiological function of hGIIA [64]. 

Recent research by Dacheux et al. [65] highlights that hGIIA’s role in host defense extends beyond anti-bactericidal properties to anti-malarial properties. Other sPLA_2_s human group IIF, hGV and hGX hydrolyze phospholipids present in lipoproteins producing non-esterified fatty acids such as polyunsaturated fatty acids that are highly toxic to the malaria pathogen, *Plasmodium falciparum*, however these sPLA_2_s are not detected in the plasma of infected patients [65]. Conversely, hGIIA has low affinity for these lipoproteins, but more effectively hydrolyses their oxidized counterparts, which are present in higher concentrations in plasma from malaria patients [65]. hGIIA is up-regulated in the plasma of patients with malaria, catalyzes these oxidized phospholipids present in lipoproteins, promoting its parasite-killing effect [65]. This effect was confirmed with the injection of recombinant hGIIA in *P. chabaudi* infected mice [65], which resulted in a reduction of peak parasitemia with increased plasma peroxidation.

### 3.2. hGIIA Function in Inflammation

The known role of hGIIA in inflammatory pathology is primarily a result of its function as an ‘amplifier of inflammation’ through generation of eicosanoids and lysophospholipids such as LPA. In physiological inflammation, hGIIA is stored in secretory glands and vesicles of innate immune cells including mast cells, platelets and eosinophils [66], prior to release as part of the inflammation response. Under initial inflammatory stimuli, cytokines such as tumor necrosis factor (TNF), interleukin-1 beta (IL-1β) and interleukin-6 (IL-6) are secreted from macrophage and mast cells, which in turn activates hGIIA expression and secretion in a range of cells including macrophage, mast cells, glial cells, platelets, astrocytes and epithelial cells. hGIIA acts through multiple positive feedback loops to drive the progression of inflammation from resolvable to chronic. Multiple reports by Triggiani and Granata et al. [67,68,69] have shown that secreted hGIIA can also bind to lung macrophage M-type receptor, although hGIIA binds weakly to this receptor relative to other sPLA_2_s. Receptor binding triggers the further cytokine gene transcription for TNF and IL-6 [67,68], as well as the release of lysosome enzyme β-glucuronidase and endothelial growth factors [69]. Through this positive feedback loop, hGIIA and cytokines amplify each other’s expression, which contributes to the so-called “cytokine” and “eicosanoid” storm [70] characteristic of severe inflammatory disorders.

The cytokine/eicosanoid amplification cycle also involves cross-talk between hGIIA and the AA-selective intracellular enzyme cPLA_2_-α. TNF increases both hGIIA and cPLA_2_-α expression in epithelial cells. Once expressed, hGIIA activates the NF-κB pathway [71,72], modulating its own expression [73]. NF-κB is also activated by hGIIA metabolite prostaglandin E_2_ (PGE_2_) via GPCR binding [74], again causing an increase in both cyclooxygenase-2 (COX-2) and eicosanoid production.

In addition to modulating intracellular signaling pathways, hGIIA also targets extracellular vesicles (EVs), which are generated from all cell lineages after cytokine activation and contain a range of cargo. EVs have a higher composition of anionic phospholipids than cell membrane which favors hGIIA activity [75]. Platelet-derived EVs (known as microvesicles) contain cyclooxygenase-1 (COX-1) and 12-lipoxygenase (12-LOX). These enzymes further metabolize the AA released from hGIIA to produce primarily thromboxane and 12-HETE which are responsible for platelet aggregation and EV internalization into neutrophils [76]. LPA derived from hGIIA enzymatic activity on EVs generated by neutrophils also initiate NF-κB signaling, binding to GPCRs and initiating PI3K/Akt pathways [77] as well as increasing cytosolic calcium levels required for cPLA_2_-α translocation to the membrane and subsequent activation.

During immune cell activation and tissue damage, mitochondria are also expelled into the extracellular space, and become another substrate for hGIIA. The outer membrane of extracellular mitochondria is hydrolyzed releasing mitochondrial DNA and other damage-associated molecular patterns (DAMPs), thereby promoting activation of innate immune cells via Toll-like receptor (TLR) signaling, again contributing to inflammation amplification [78,79]. 

#### 3.2.1. hGIIA in Neuroinflammation

Chronic neuroinflammation is associated with and a biomarker for major neurodegenerative diseases including Alzheimer’s, Parkinson’s disease and multiple sclerosis. The importance of studying the function of PLA_2_ enzymes in the central nervous system has been clear since the earliest days of the field [14,15]. The potential role(s) of mammalian group IIA PLA_2_, within a broader discussion of the role of lipid mediators and potential roles for PLA_2_ enzymes in neuroinflammation in the central nervous system (CNS) were well reviewed in the mid-late 2000s [80,81,82,83]. Of note was a study that characterized the expression profile of multiple sPLA_2_ isoforms in the rat CNS [84]. sPLA_2_-IIA was expressed in the brainstem and spinal cord, specifically the cervical, thoracic and lumbar segments, the spinal trigeminal and the facial motor nuclei and dorsal-and ventral horns of the spinal cord. Mechanistically, early research by Hernandez et al. [85,86] has identified that hGIIA initiates both calcium and extracellular receptor kinase (ERK) signaling in astrocytoma cell lines through binding to a PLA_2_ receptor on the cell surface, a mechanism independent of hGIIA catalytic activity. Both of these signaling pathways result in activation of cPLA_2_-α and increased eicosanoid production. In a later study [87], hGIIA was implicated in the cytotoxicity of cytokine-stimulated THP-1 and human primary astrocytes towards the human neuroblastoma cell line SH-SY5Y via a non-enzymatic mechanism. 

Since that time, there has been little work reported specifically on sPLA_2_-IIA or hGIIA function in the CNS. Studies have concentrated on characterizing lipid mediator profiles in neuroinflammation [88,89,90]. While the evidence cited above suggests further investigation of the role of hGIIA in neuroinflammation is warranted, the tools needed to robustly evaluate hGIIA in the setting of neuroinflammation are lacking. These would necessarily include the use of genetically modified animal models that allow for conditional and tunable over-expression of sPLA_2_-IIA in specific target cell types. For these studies to be translatable to human disease, methods of selectively and specifically modifying hGIIA function by pharmacological means in a non-toxic way are also essential. Such approaches have not yet been identified or robustly tested in neuroinflammation.

#### 3.2.2. hGIIA in Asthma/COVID-19

Since the discovery that the slow reacting substance of anaphylaxis was a leukotriene AA metabolite [91], the pathway has been of interest to asthma and allergy researchers. hGIIA became of interest when it was established that expression of AA and hGIIA is increased in the bronchoalveolar lavage (BAL) of patients with asthma after antigen inhalation challenge [92]. hGIIA is one of the major sPLA_2_s responsible for enzymatic activity in the bronchoalveolar lavage fluid [93]. Furthermore, hGIIA released from mast cells activates human lung macrophages via paracrine pathways, again progressing inflammation associated with asthma and other respiratory disorders [94]. The role of hGIIA in asthma, however, is not as clear as it is for the related secreted PLA_2_ hGX [95,96], for which there is evidence from gene deletion studies in mice that the enzyme is functionally important in both acute and chronic asthma models. Equivalent experiments with sPLA_2_-IIA are, to the best of our knowledge, yet to be performed. 

Recent data has highlighted a new link between hGIIA and SARS-CoV-2 infection during the COVID-19 pandemic [97]. Out of 80 clinical indices measured, including age and body mass index, hGIIA and blood urea nitrogen (BUN) were the strongest predictors of severe disease, with a decision tree based on these two indicators being able to stratify patients with mild severe or fatal infection on a validation cohort of COVID-positive patients. Sars-CoV-2 infection also alters the lipidome, resulting in higher concentrations of lyso-PE and lyso-PS, with no changes in lyso-PC, which is a hallmark of hGIIA activity [97]. Other research suggests COVID-19 may also increase levels of PS on the surface of the cell membrane, exposing cells to catalytic activity of hGIIA [97,98]. The authors suggest that hGIIA may mediate severe COVID-19 disease and as its expression is closely linked to disease outcome, that hGIIA is an attractive therapeutic target. Correlation of hGIIA expression with severity has been demonstrated in children with COVID-19 with highest levels in those with acute multisystem inflammatory syndrome [99].

#### 3.2.3. hGIIA in Atherosclerosis

hGIIA has long been proposed as a promoter of atherosclerosis, since it was first identified as being expressed in, atherosclerotic plaque and the dendritic cell marker CD1a-positive cells in particular [32]. Preclinical cell culture and in vivo studies supported this view leading to a sophisticated model of hGIIA function [100]. For example, hGIIA increased levels of 12-LOX and 15-LOX, which oxidize low density lipoproteins (LDL) [101], transforming them into a substrate for hGIIA [102]. Once LDL phospholipids are metabolized by hGIIA they become small dense LDL particles which are pro-atherogenic [103]. Further expression studies showed the enzyme was highly expressed in human atherosclerotic lesions colocalizing with macrophage and smooth muscle cells [102]. It became clear that elevated serum hGIIA is an indicator of coronary artery disease and its concentration an indicator of the risk of coronary events [104], making it not only a prognostic marker but also a potential therapeutic target. These data, supported by preclinical efficacy studies in animal models of atherosclerosis, provided the impetus for a double-blind placebo-controlled trial of Varespladib (500 mg daily) recruiting 5145 acute coronary syndrome patients [34]. This trial was unexpectedly terminated due to early signals of increased risk of myocardial infarction (MI) in the treatment group, effectively ending the development of this drug as a treatment for cardiovascular disease (CVD).

Whether these data rule out hGIIA as a target for the treatment of CVD remains a matter of debate. Whether increased risk of MI on Varespladib treatment is related to inhibition of sPLA_2_ activity or due to unknown off-target effects of the drug is not likely to be known. As mentioned above, Varespladib is non-selective for hGIIA, being a potent inhibitor of group IIE, hGX, hGV and group IB sPLA_2_. There are significant differences in substrate specificity among these enzymes with group X and group V sPLA_2_ having higher catalytic activity for phosphatidylcholine, the major phospholipid component of lipoproteins. As described above, hGIIA has greater catalytic activity on oxidized lipoproteins, indicating hGIIA targets a substantially different population of lipoprotein substrates than group V or X sPLA_2_. While oxidative stress is an important contributor to atherosclerosis, and oxidized LDL is a well-established proatherogenic modification, there are no definitive studies that robustly determine the effect of hGIIA catalysis of oxidized LDL on atherogenesis. It is difficult to explore the role of hGIIA pharmacologically in the absence of sPLA_2_ isoform-selective inhibitors. Without substantially more robust data supporting a role for hGIIA and defining the precise mechanism of action of the enzyme within the context of CVD, it is unlikely that hGIIA will be pursued as a target for therapy.

#### 3.2.4. hGIIA in Sepsis

The first clinical study to determine serum PLA_2_ activity, subsequently shown to be hGIIA activity, in any disease established an association between this activity and severity of disease in sepsis and septic shock [18]. This relationship is robust with hGIIA being recognized as a key biomarker and predictor of mortality in this disease [105,106]. In addition, a single study establishes that hGIIA is also an important marker in combination with procalcitonin in predictively distinguishing patients with sepsis from patients with noninfectious systemic inflammatory response syndrome on presentation at Emergency Departments (ED) [107]. Despite evidence of benefit in preventing misdiagnosis and consequent mistreatment of patients on presentation to ED, translation of these findings into a clinically useful test format available for widespread affordable use remains the major challenge for this approach.

hGIIA function during sepsis can be best described as a double-edged sword. The well-known antibacterial function of hGIIA coupled with high serum and tissue concentrations in severe cases signify its critical role in host defense in clearing infection, yet its catalytic activity is also directed towards clearing apoptotic cells including extracellular mitochondria and the membranes of apoptosing cells. The resulting DAMPs contribute to the cytokine storm and deregulated hyperinflammation [78,79] that contributes to dysregulation of tissue function that leads to hypotension and organ failure. A placebo controlled clinical trial has been conducted aimed at evaluating the efficacy of hGIIA inhibition in patients with at least two sepsis-induced organ failures treated with Varespladib (LY315920NA/S-5920) by continuous intravenous infusion for 168 h at a target plasma concentration of 800 ng/mL [108]. The trial was terminated following examination of data on 250 patients indicated no improvement in 28-day all-cause mortality could be expected. The negative trend in 28-day all-cause mortality was most pronounced in patients with cardiovascular at baseline and patients with negative blood cultures at baseline. These findings call into question the utility of inhibiting hGIIA activity in severe disease. Interestingly, of the other sPLA_2_ enzymes known to be inhibited by Varespladib, group V sPLA_2_, is not detectable in serum in patients and there is very little, if any, data on the expression of group X sPLA_2_.

#### 3.2.5. hGIIA in Rheumatoid Arthritis

Discovering the importance of hGIIA in arthritis was always an important scientific goal since it was first purified from synovial fluid of patients with RA [19,20,21,23]. Measurements of hGIIA in serum established a correlation between hGIIA concentration and disease severity, demonstrating that serum hGIIA could be used as an indicator of the activity of disease [28,109]. The mechanism of action of hGIIA was subsequently explored in fibroblast-like synoviocytes from RA patients [110]. Exogenous hGIIA, though inactive on unstimulated cells, was shown to amplify cytokine-mediated induction of prostaglandin production by an intracellular, catalysis-independent mechanism that upregulated expression of the cPLA_2_-α-cyclooxygenase-2 pathway.

Eicosanoid upregulation was the result of increased cPLA_2_-α activity however the precise mechanism of how hGIIA induces this has not been fully elucidated. Induction was accompanied by rapid internalization of hGIIA and colocalization with the intermediate filament protein vimentin [111]. Pharmacological disruption of this interaction suppressed hGIIA-dependent PGE_2_ production. Exogenous hGIIA was also capable of AA release in these experiments, however, the substrate for this release was extracellular vesicles (EVs). The pro-inflammatory role of hGIIA in RA was confirmed by genetic deletion studies in an adoptive transfer mouse model of arthritis [40]. Deletion of the hGIIA gene resulted in suppression of arthritis, while, surprisingly, deletion of group V sPLA_2_ exacerbated joint inflammation. Thus hGIIA has a bifunctional role in modulating RA synoviocyte AA metabolism and the chronic inflammation driving arthritis not only through its enzymatic activity on EVs [112], but also through amplifying intracellular eicosanoid production via a catalytically independent mechanism.

Inhibition of sPLA_2_ has been evaluated in a double-blind placebo-controlled clinical trial of an oral formulation of Varespladib (LY333013) in 251 active RA patients not responsive to concomitant disease modifying drugs (DMARDs). Patients received doses ranging from 50 to 1000 mg or placebo once daily for 12 weeks [113]. Though well tolerated, with dose-response relationships reported for ACR20 responses (a 20% reduction in severity scores) and C-reactive protein at 1 week, these signals were lost in subsequent weeks due to ACR20 response increasing in all groups including placebo, leading investigators to conclude the treatment was ineffective in combination with DMARD treatment. Though not known at the time, the lack of selectivity for hGIIA over group V and group X sPLA_2_ likely confounds this study in the light of the anti-inflammatory effects of group V sPLA_2_ identified in the gene deletion studies [40].

#### 3.2.6. hGIIA in Cancer

The discovery of elevated expression of hGIIA in a range of cancer tissues and in serum from cancer patients [114,115], sparked interest in its role in cancer progression, a subject that has been extensively and regularly reviewed. [64,116,117,118,119,120]. A protumorigenic role for hGIIA is exemplified in prostate cancer because exogenous hGIIA promotes proliferation in cultured cancer cells and inhibition of hGIIA function slows tumor growth in xenograft models of disease [121]. hGIIA expression is increased in multiple cancers. However, the effect of aberrant expression varies with the tissue site of the tumor (Table 1). In prostate cancer, hGIIA’s pro-tumorigenic effect is likely mediated through production of LPA and its ability to upregulate cPLA_2_-α mediated eicosanoid production. It is well established that both LPA and eicosanoids are heavily involved in carcinogenesis and control of the tumor microenvironment through interactions with GPCRs, as reviewed by Mills and Moolenaar [122] and Wang and Dubois [123], respectively.

As one function of hGIIA is a catalytically independent activation of cPLA_2_-α [126], it is hypothesized that this mechanism of action is the same as that proposed in arthritis, with vimentin again implicated as a binding partner of hGIIA [61]. Further investigation is currently underway to identify the possible role of vimentin in hGIIA’s carcinogenic effect, and whether inhibition of this proposed interaction could be used for therapeutic treatment. No clinical trials have yet been published following administration of an hGIIA inhibitor to cancer patients. 

## 4. hGIIA Mechanism of Action

All known functions of hGIIA are driven either by its activity as a PLA_2_ (catalysis-dependent) or through cell signaling and interactions with other proteins (catalysis-independent) mechanisms. Catalytically inactive mutants of hGIIA have been previously used to determine the mechanism of action in multiple pathologies.

### 4.1. Catalysis-Dependent Mechanisms

hGIIA exhibits the same highly conserved catalytic dyad as other PLA_2_s, consisting of a histidine-48 and an aspartate-99 in close proximity. There are two proposed models for catalytic function: the ‘triad’ model [127] and the ‘calcium-coordinated oxyanion’ model [33,128]. As discussed by Kim et al. [64], the calcium-coordinated oxyanion model requires a lower activation energy and is more consistent with experimental data from point substitutions. This model also proposes a role for the calcium binding loop in the catalytic action of hGIIA, therefore explaining the requirement for millimolar calcium concentration observed under experimental conditions [22]. The area of hGIIA that directly binds to phospholipids known as the interfacial binding surface (i-face) consists mostly of hydrophobic amino acids, as well as cationic residues that contributes to the specificity of hGIIA for anionic substrates. The i-face is shaped to allow a single phospholipid entry to the active site for hydrolysis by the catalytic dyad. The absence of a tryptophan on the i-face also drives substrate specificity, as confirmed by tryptophan substitution [129]. Unlike hGIIA, the amphiphilic indole moiety of tryptophan is present in other sPLA_2_s with non-selective substrate specificity and facilitates penetration into the lipid interface of the phospholipid bilayer, increasing the access of the catalytic site to zwitterionic PC substrate. The absence of tryptophan explains, in part, the relatively weak catalytic activity of hGIIA on PC substrates. 

The catalytic function of hGIIA is largely confined to extracellular locations, due to the requirement for millimolar calcium concentrations. Biological substrates for hGIIA catalysis such as bacteria, EVs, extracellular mitochondria and oxidized LDL are hydrolyzed in the extracellular space. Though hGIIA catalytic activity is likely to be involved in chronic inflammation, atherosclerosis, sepsis and respiratory-related pathologies, there are few robust experimental models that can test the relative importance of catalysis in vivo. However, the development of lipidomics approaches [130], coupled with the increasingly versatile methods of in vivo CRISPR-based genetic modification [131] provides approaches to explore the impact of hGIIA catalytic activity on the composition of lipid mediators produced in biological settings.

### 4.2. Catalysis-Independent Mechanisms

It is well-established that hGIIA function is not limited to its catalytic activity. Many of the observed effects of hGIIA on biological systems, particularly in pathological settings characterized by persistent aberrant expression of hGIIA, may be attributed to direct or indirect perturbation of intracellular cell signaling pathways mediated through protein-protein interactions.

The catalytically-independent upregulation of eicosanoid production has been observed in cell culture models of several disorders including arthritis, neuroinflammation and cancer [110,126,132]. In rheumatoid synoviocytes, upregulation of PGE_2_ production by exogenously-applied hGIIA requires cytokine-mediated activation of the cPLA_2_-α/COX-2 biosynthetic pathway. Although hGIIA alone upregulates the expression of COX-2 and can activate ERK phosphorylation, additional signals from TNF, likely through cPLA_2_-α and NF-κB activation, are required to increase PGE_2_ production. In combination, hGIIA amplifies TNF-mediated PGE_2_ production by further upregulating steady-state COX-2 protein expression and this effect is independent of hGIIA catalysis [110,133]. Other studies in astrocytoma cells suggest that PGE_2_ upregulation is a result of hGIIA’s ability to increase cPLA_2_-α function. In this case, evidence suggests that exogenous hGIIA binds to a PLA_2_ receptor (PLA_2_R) on the cell surface which initiates activation of two separate pathways; the MAP kinase cascade which activates cPLA_2_-α, and activation of PLCγ and release of calcium from intracellular stores [85,86]. This effect is not mitigated with the addition of *p*-bromophenacyl bromide (BPB) which halts enzymatic activity of hGIIA, confirming that this mechanism is independent of catalysis. Subsequent research indicates ERK signaling is initiated through phosphorylation of the endothelial growth factor receptor (EGFR) [132]. This mechanism of action also occurred in breast cancer cell lines and was confirmed by Dong that hGIIA acts as a ligand, directly binding to and activating EGFR [73,134]. Interestingly, hGIIA structural similarities with EGF likely drive the direct binding with the receptor, rather than its positive charge [73]. Stimulation of EGFR subsequently initiates human epidermal growth factor/human epidermal growth factor 2 (HER/HER2) signaling and rat sarcoma (RAS)/rapidly accelerated fibrosarcoma (RAF)/ERK kinase (MEK)/extracellular-signal-regulated kinase (ERK) signaling, leading to cPLA_2_-α activation. hGIIA also activates macrophages via binding to M-type receptors on the surface, stimulating the release of cytokines TNF and IL-6 [68].

Other proteins have been identified to interact with hGIIA in cell culture models (reviewed in Kim et al. [64]). In human embryonic kidney 293 (HEK293) cells, heparan sulfate proteoglycans (HSPGs) present on the cell surface also bind hGIIA through charge interactions [135]. In these cells, HSPGs are required for hGIIA sequestration and for hGIIA-driven eicosanoid over-production [136]. Cell-surface integrins αvβ3 and α4β1 also bind hGIIA via an allosteric mechanism [137].

Vimentin is an intracellular intermediate filament cytoskeleton protein that is also detectable outside cells under conditions of stress and is reported to bind hGIIA. The vimentin knock-out mouse has no obvious phenotype unless animals are subjected to stress, and it is clear that vimentin is involved in cellular stress responses [138]. hGIIA binds to extracellular vimentin in apoptosing T cells [139], and colocalizes with intracellular vimentin in FLS cells [111] As discussed by Kim et al. 2020 [64], vimentin binds cPLA_2_-α and ERK in a calcium dependent manner [140,141], both proteins that are required for catalytically-independent upregulation of eicosanoid production. Vimentin may act as an adaptor molecule, facilitating further interactions of these proteins, as inhibition of the hGIIA/vimentin interaction results in a reduction of hGIIA-generated eicosanoids [111]. Further research is required to elucidate this possible mechanism of action to define the role for vimentin in eicosanoid production.

**Figure 2:**
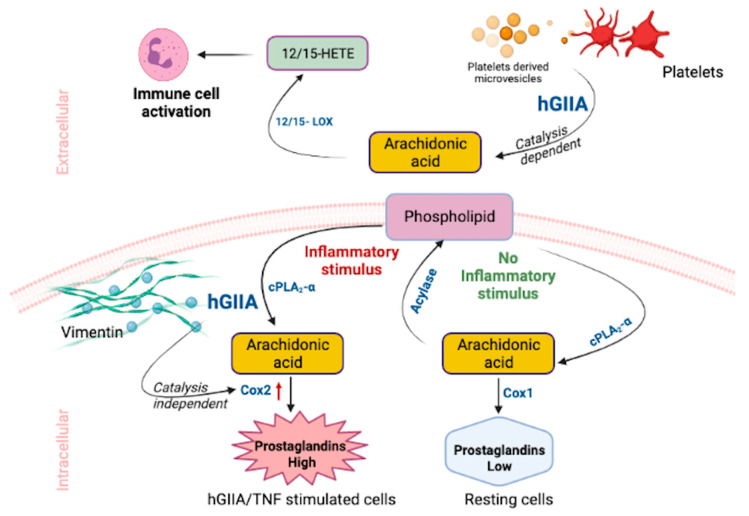
hGIIA catalysis-dependent and -independent functions in inflammation.

A model of the site-specific mechanism of action of hGIIA is shown in Figure 2. hGIIA function is compartmentalized in vivo due to the requirement for millimolar calcium concentrations for optimal hGIIA enzyme activity that restrict its catalytic activity primarily to the extracellular environment. Catalysis of phospholipid substrates is driven by hGIIA affinity for substrate PA > PE~PG > PS >> PC in physiological conditions facilitating clearance of bacteria in barrier secretions and in the innate immune response. In pathological inflammation, hGIIA catalysis indirectly contributes to immune cell activation through the release of AA from extracellular vesicles, which is further metabolized by 12/15 LOX enzymes to generate activating DAMP ligands that contribute to innate immune responses through TLR4 activation. Aberrant upregulation of hGIIA in response to inflammatory cytokines results in hGIIA internalization and colocalization with the intermediate filament protein vimentin in mesenchymal cells. Internalization results in catalysis-independent upregulation of COX-2 and subsequent overproduction of PGE_2_ relative to that seen in resting cells where AA released by cPLA_2_-α is re-esterified back into phospholipids via the Lands cycle through the action of acylases.

## 5. Pharmacological Inhibition of hGIIA Function

Considering the role of hGIIA in physiology and its dysregulation associated with multiple disorders, it is clear that identification of potent inhibitors of hGIIA may be useful not only in alleviating disease symptoms, but also in defining hGIIA function. Natural and synthetic inhibitors of hGIIA have been identified. However, as described above, no hGIIA inhibitors have currently been approved due to toxicity concerns. Inhibitors of hGIIA have been previously reviewed [64,142]. Originally hGIIA inhibitors were classified based on their specific chemical conformations but recently a functional basis of classification was introduced by Lee et al. [111], viz. inhibitors which are selective for (a) the catalysis-dependent mechanism or (b) the catalysis-independent mechanism or (c) dual function inhibitors.

### 5.1. Inhibitors of the Catalysis-Dependent Mechanism

BPB is the only confirmed catalysis-dependent inhibitor of hGIIA. It is a chemically synthesized molecule shown to abrogate the enzymatic ability of hGIIA through alkylation of the active-site histidine without affecting hGIIA non-catalytic function. However, while useful for inhibiting the activity of purified hGIIA in exogenous addition experiments in vitro, this compound is a non-selective alkylating agent, so therefore not selective for hGIIA function on in vivo administration [143]. Nonetheless its mode of binding provides insight into how selective hGIIA catalysis inhibitors may be designed [111]. 

There are several reports of naturally-occurring compounds that modulate hGIIA catalytic function, however their selectivity for hGIIA and their precise mechanism of action is unclear. For example, the anti-inflammatory activity of a natural pentacyclic triterpenoid, maslinic acid, is reported to be due to its ability to bind to the calcium binding loop and i-face of hGIIA thereby reducing the release of fatty acids and lysophospholipids [144] Interestingly, maslinic acid was also found to significantly reduce lipid accumulation in macrophages by suppressing hGIIA-induced endocytic activity. This, in turn, inhibits LDL uptake in foam cells and suppresses hGIIA-induced up-regulation of PGE_2_ levels, inhibiting monocyte migration and differentiation an immune-inflammation processes [145]. Sulforaphane [145], orientin [146], vicenin-2 [147] and scolymoside [148] are other naturally-occurring compounds that inhibit the in vitro and in vivo enzymatic activity of hGIIA in LPS stimulated human umbilical vein endothelial cells (HUVECs) or in a cecal ligation and puncture, an in vivo model of sepsis. These natural compounds are likely to have pleiotropic effects on several pathways in addition to hGIIA inhibition that could explain these effects. For example, maslinic acid is known to bind to multiple target proteins associated with malarial infection [149]. Their utility as pharmacological agents to probe the mechanism of action of hGIIA is therefore limited. In addition, the contribution of observed hGIIA inhibition to their anti-inflammatory action relative to other effects is difficult to establish.

### 5.2. Inhibitors of the Catalysis-Independent Mechanism

A tryptic digestion product of hGIIA, (^70^FLSYK^74^) was found to be a weak inhibitor of hGIIA catalytic activity [150]. Structure-activity studies coupled with cyclization improve the potency of the compound through replacement of the aromatic amino acids phenylalanine and tyrosine with 2-naphthylylalanine and lysine with arginine [151]. The resulting compound cyclo-((2-Nal)-Leu-Ser-(2-Nal)-Arg), known as c2, selectively inhibits exogenous hGIIA-mediated catalysis-independent PGE_2_ synthesis with weak inhibition of catalytic activity relative to active site inhibitors Me-Indoxam, KH064 and LY311727 [111]. The active site inhibitors also inhibited the catalysis-independent function of hGIIA with similar potency to the peptides, demonstrating that these compounds are dual-function inhibitors. The defining feature of dual function inhibitors is that, in addition to tightly binding to the active site of hGIIA, they also protrude from the hydrophobic channel that allows substrate entry to the active site, thus disturbing the i-face of hGIIA. The c2 peptide acted as an inhibitor of the interaction between hGIIA and vimentin that occurs on internalization of hGIIA into the synovial cells [111]. This finding supports a model that hGIIA-dependent amplification of PGE_2_ production through upregulation of COX-2, also inhibited by c2, is mediated by an hGIIA-vimentin interaction. These peptides have been shown to slow the growth of prostate tumors in xenograft mouse models of prostate cancer [121].

Peptide inhibitors of the binding of hGIIA to integrin ανβ3 have also been developed from peptide libraries, and a tetrapeptide molecule linked with pyrazolylthiazole moiety was found to have IC_50_ of 20 µM in a cell adhesion assay using immobilized hGIIA [152]. The docking simulation, as part of this study, predicted that this inhibitor interacts with Arg81 and Arg108 of hGIIA, rather than to the integrin [152]. Whether these compounds have efficacy in in vivo models of inflammation remains to be determined.

## 6. Summary and Conclusions

The last 30 years of research has demonstrated the complexity of PLA_2_ function with the discovery of a superfamily of PLA_2_ enzymes. The secreted enzymes have diverse and often organ-specific roles in regulating eicosanoid metabolism. hGIIA catalytic activity is clearly associated with the immune response to both bacterial and possibly viral infection and its expression under physiological conditions is limited to glandular secretions with barrier function and to innate immune cell granules. Under conditions of cellular stress, platelets and cells release EVs enriched in hGIIA substrates which are acted on by hGIIA in concert with lipoxygenase enzymes to produce lipid mediator DAMPs that activate cellular immunity through TLR signaling. We have characterized an intracellular catalysis-independent mechanism whereby hGIIA superinduces cytokine-mediated eicosanoid production through colocalization with vimentin and induction of COX-2 expression. The molecular mechanism of induction is not clearly-defined. However the effect is blocked by cyclic peptide inhibitors that interfere with hGIIA-vimentin colocalization. hGIIA also binds to other proteins. Nevertheless, the role of these interactions in pathology is yet to be determined.

The failure of clinical trials with potent inhibitors of hGIIA catalysis due to unexpected safety signals, has effectively halted further development of this class of inhibitor for the treatment of inflammation. It remains an important goal to determine a mechanism that can explain these toxicity signals. It is equally important to concentrate effort on developing both structurally-selective inhibitors that have minimal activity on other secreted PLA_2_s and functionally selective inhibitors that are selective for hGIIA catalysis and inhibitors that are selective for hGIIA catalysis independent function. Structure-based drug design approaches continue to offer the possibility of discovering inhibitors with improved selectivity for catalysis as demonstrated for by the identification of an indole inhibitor with 100-fold and 50-fold greater potency for group IIE sPLA_2_ over group IID and hGX respectively [42,153]. However, selective inhibitors of hGIIA catalysis remain elusive. Further, thus far, catalysis inhibitors, with the exception of BPB, also inhibit the catalysis-independent function of hGIIA [111]. hGIIA binds to several different proteins [63], so it will be important to learn more about the functional consequences of these interactions and to identify compounds that can selectively block pathological interactions. 

The complexity of hGIIA expression and function is ideally suited to the application of new technologies such as CRISPR/Cas9-mediated gene modification to create precision models to determine the effect of ablation of hGIIA enzyme activity in cellular and animal models of disease. Additionally, dramatic advances in omics technologies resulting in accessible and reliable generation of lipidomics, proteomics and transcriptomics data allow detailed examination of the effect of manipulation of hGIIA function on global metabolism. This coupled with emerging advances in network pharmacology approaches [154,155] will enable the integration of complex omics data as well as known biochemistry of the metabolic pathways and cellular interactions into testable in silico models to assess the system-level effect and efficacy of hGIIA inhibitors in different cellular and tissue contexts. Further, advances in imaging techniques coupled with omics technologies in both physiological and pathological states [156,157,158] have created opportunities to examine the dynamic intracellular organization of hGIIA and its protein interaction partners to explore the temporal and spatial organization of the enzyme in cellular and tissue contexts. 

The complexity of the role and function of hGIIA in pathology is an ideal and timely challenge that is addressable by these recent technological advances. These advancements are likely to define the relevance of hGIIA function in pathological settings and provide guidance on how best to pharmacologically intervene to limit the proinflammatory effect of hGIIA without toxicity. 

## Figures and Tables

**Table 1:** hGIIA expression and prognostic association in cancer.

Cancer Type	Expression	Association with Prognosis	Reference
Prostate	Increased	Shorter Patient Survival	[118]
Breast	Increased	Shorter Patient Survival	[118]
Gastric	Increased	Leading to Longer Patient Survival and Less Metastasis	[118]
Lung	Increased	Shorter Patient Survival	[118]
Oesophageal	Increased	Unknown	[118]
Colon	Varies from Site	Protective	[119]
Liver	High	Shorter Patient Survival	[119]
Brain	High	Shorter Patient Survival	[119]
Pancreatic	High Early Stage	Better Prognosis	[119]
	Low Late Stage	Poor Prognosis	[119]
Ovarian	High	Shorter Patient Survival	[119]
	Decreased	Following Chemotherapy	[124]
Skin	Increased		[125]

## Data Availability

Not applicable.
